# The 10-year anniversary of the dissemination and implementation models in health interactive webtool: History, refinement, use, and guidance

**DOI:** 10.1017/cts.2025.10128

**Published:** 2025-08-22

**Authors:** Borsika A. Rabin, Bryan Ford, Rachel G. Tabak, Rebekah Gomes, Kera N. Swanson, Sara Malone, Maggie Padek Kalman, Ross C. Brownson, Russell E. Glasgow

**Affiliations:** 1 Herbert Wertheim School of Public Health and Human Longevity Science, University of California San Diego, La Jolla, CA, USA; 2 UC San Diego Altman Clinical and Translational Research Institute Dissemination and Implementation Science Center, University of California San Diego, La Jolla, CA, USA; 3 University of Colorado Anschutz Medical Campus, Aurora, CO, USA; 4 Prevention Research Center, School of Public Health, Washington University in St Louis, St Louis, MO, USA; 5 Health Design and Impact Lab, School of Public Health, Washington University in St Louis, St Louis, MO, USA; 6 Frontiers Clinical and Translation Science Institute, University of Kansas Medical Center, Shawnee Mission Parkway, Fairway, KS, USA; 7 Alvin J. Siteman Cancer Center, Washington University School of Medicine, Washington University in St Louis, St Louis, MO, USA

**Keywords:** Dissemination, implementation, framework, guidance, webtool

## Abstract

Theories, models, and frameworks (TMFs) are essential tools in dissemination and implementation (D&I) research, yet selecting and applying the most appropriate TMF is routinely a challenge, particularly for those new to the field. To address this need, we developed the *Dissemination and Implementation Models in Health* webtool (www.dissemination-implementation.org) a free, interactive, and evolving online resource designed to support the thoughtful use of D&I TMFs across all phases of research and practice – from planning through assessment. Created through a multi-institutional collaboration and refined using human-centered design, the webtool includes features such as logic model development, D&I TMF selection and comparison, guidance on combining and adapting models, strategies for application, and linkages to measurement tools. Since its initial release in 2014, the webtool has expanded to include over 110 D&I TMFs and new thematic content areas, including a section dedicated to health equity. It can be used in D&I trainings, proposal development, consultations, and academic coursework. Usage analytics and community feedback reflect ongoing relevance, utility, and evolving needs. The webtool continues to address a significant gap in D&I infrastructure by guiding users in selecting and operationalizing D&I TMFs, ultimately supporting more rigorous, context-sensitive translational research and practice.

## Background

Dissemination and implementation (D&I) science has been a recognized area of health research for over two decades. A central tenet of D&I research is the early, ongoing, and meaningful use of theories, models, and frameworks (TMFs) that can inform the planning, operationalization, evaluation, sustainment, and dissemination of research projects and their products. The D&I field offers a vast array of TMFs, making selection and use a challenging task for researchers and practitioners. In our experience, one of the most frequently asked questions in consultations for D&I research is *“How do I select and apply an appropriate D&I science TMF?.”* The importance of D&I TMFs is further emphasized by the expectation that D&I science grant proposals and paper submissions include a well-operationalized D&I TMF [1]. The proper use of a D&I TMF is listed as one of the ten key ingredients of successful (funded) D&I proposals [1]. In contrast, the lack of a well-operationalized D&I TMF or its inappropriate specification or use is a common reason that proposals are scored poorly [1,2].

To address this need, we developed an interactive webtool to provide guidance to researchers and practitioners – both newer and more experienced in the field of D&I. The Dissemination and Implementation Models in Health (D&I Models in Health) (www.dissemination-implementation.org) is an interactive, online resource designed to help researchers and practitioners navigate D&I TMFs through planning, selecting, combining, adapting, using, and linking to assessments for D&I TMF application. It also serves as a valuable resource for numerous D&I trainings and courses, and as a tool for experienced researchers to identify the D&I TMF(s) that more appropriately aligns with their project. Examples where the webtool has been utilized include workshops and live demonstrations at the AcademyHealth Annual Conference of the Science of Dissemination and Implementation in Health and at the Society of Implementation Research Consortium biannual meeting, graduate courses with subsequent mentoring sessions at UC San Diego, Washington University in St Louis, and the University of Colorado, and fellowship training at the University of Colorado.

The webtool was designed to help researchers and practitioners develop a logic model or diagram for their research or practice question to identify the key constructs involved, and based upon this, select the D&I TMF(s) that best fit(s) their research question or practice problem. It then provides guidance on when and how to combine multiple D&I TMFs, adapt the D&I TMF(s) to the study or practice context, use the D&I TMF(s) throughout the research or practice process, and find assessments to measure the key constructs of the D&I TMF(s) selected.

In this paper we (1) describe the functions, evolution, and use of the D&I Models in Health webtool since its initial launch in 2014; (2) summarize its current content, functionality, and new sections; (3) provide guidance on the potential uses of the webtool with case examples; and (4) describe plans for future developments for the webtool.

## Overview and evolution of the D&I models in health webtool (2014–present)

### Development and history

The D&I Models in Health webtool was developed and is maintained as a collaborative effort of D&I scientists from the Adult and Child Center for Health Outcomes Research and Delivery Science Dissemination and Implementation Science Program (ACCORDS) at the University of Colorado Denver, the Prevention Research Center and the Dissemination and Implementation Research Core (DIRC) at the Washington University Institute for Clinical and Translational Science and the Altman Clinical and Translational Research Institute Dissemination and Implementation Science Center (DISC) at UC San Diego. Funding for the development of the webtool was obtained through various sources. The initial form of the webtool was developed with funding from a National Cancer Institute center grant (i.e., Centers of Excellence for Cancer Communication Research). Maintenance and follow-up expansions were supported by institutional funding through ACCORDS and the Washington University in St Louis Prevention Research Center, Center for Diabetes Translation Research, and the Institute of Clinical and Translational Sciences as well as supplemental funding from NCI through the P50 Implementation Science Centers in Cancer Control and contributed time from the UC San Diego DISC. This cross-institutional collaboration was essential for generating robust, diverse content and ensuring the financial sustainability and long-term evolution of the webtool.

**Figure 1.**
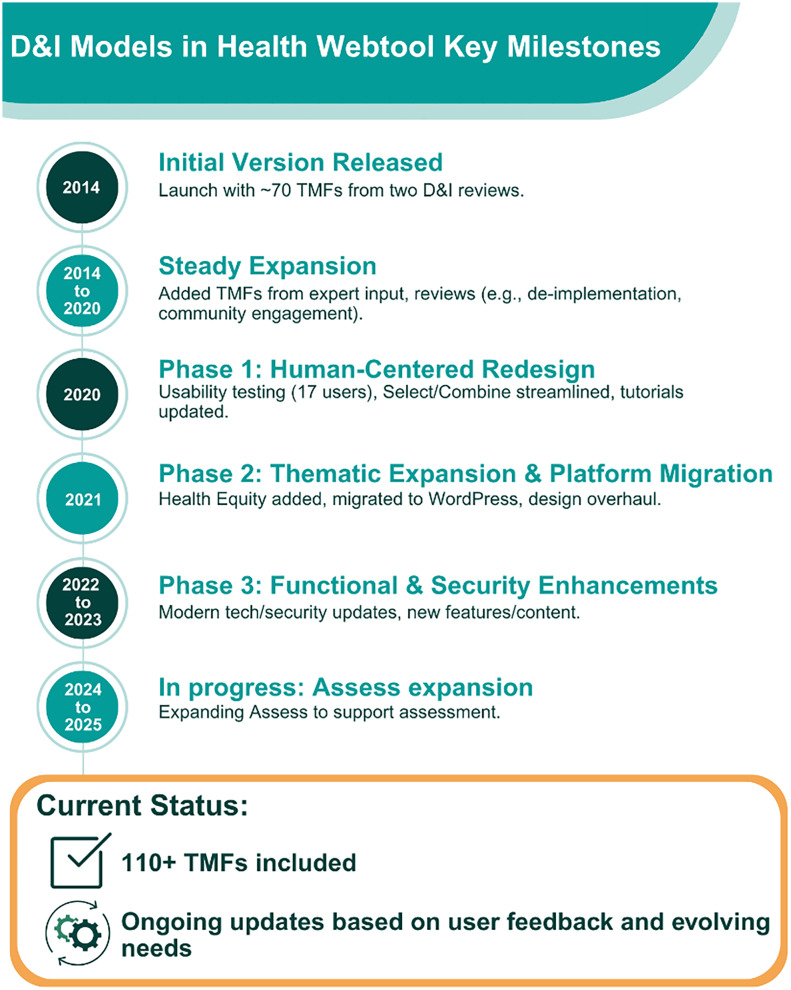
Timeline of Key Milestones in the Development of the Dissemination and Implementation (D&I) Models in Health Webtool. D&I = dissemination and implementation; TMF = Theories, Models, and Frameworks.

The key milestones in the development of the webtool are outlined in Figure 1. The initial version of the webtool was released early 2014 based on a set of D&I TMFs selected from two key reviews of the D&I literature [3,4]. Over the past decade, additional D&I TMFs were added based on expert recommendations and the availability of more recent reviews [5–8]. This includes topically focused reviews such as de-implementation TMFs [5,9,10] and community-engagement focused TMFs [11]. The criteria for including D&I TMFs are twofold: (1) the proposed addition can be defined as a theory, model, or framework (i.e., unstructured guidance documents, implementation strategies, and other entities not qualifying as a TMF are not included) AND (2) the proposed addition needs to have relevance to D&I science or practice (i.e., solely patient/client-focused behavioral theories would not qualify). The number of D&I TMFs included in the webtool increased from around 70 to over 110. This first version of the webtool included the main functions of Select, Adapt, Integrate, and Find Measures. Between 2022 and 2023, the webtool was expanded with new functionalities and content and updated to meet the latest technology and security standards [12].

Improvements to the webtool occurred in three main phases. First, in 2020, a human-centered design approach was used to update the format, function, and content of the webtool. The usability and user testing process involved 17 individuals from diverse backgrounds and varying levels of experience with D&I science, resulting in major improvements to the web tool described elsewhere [12]. Key changes in this revision included the streamlining of the process for Select, the addition of the guidance section for Combine, and revision and refinement of the tutorials including brief videos and text-based guidance. Second, in 2021, additional funding was secured to expand the webtool with special topics. During this revision, we also modernized the look and underlying programming platform for the webtool. The webtool moved from an older content management system to a more flexible and easier to maintain platform (i.e., WordPress). Third, in the most recent expansion, we are currently enhancing the Assess section to serve as a one-stop-shop ranging from planning to assessment in D&I research.

## Current Features, Content, and Structure of the Webtool

### Content and Functionalities of the D&I Models Webtool

**Figure 2.**
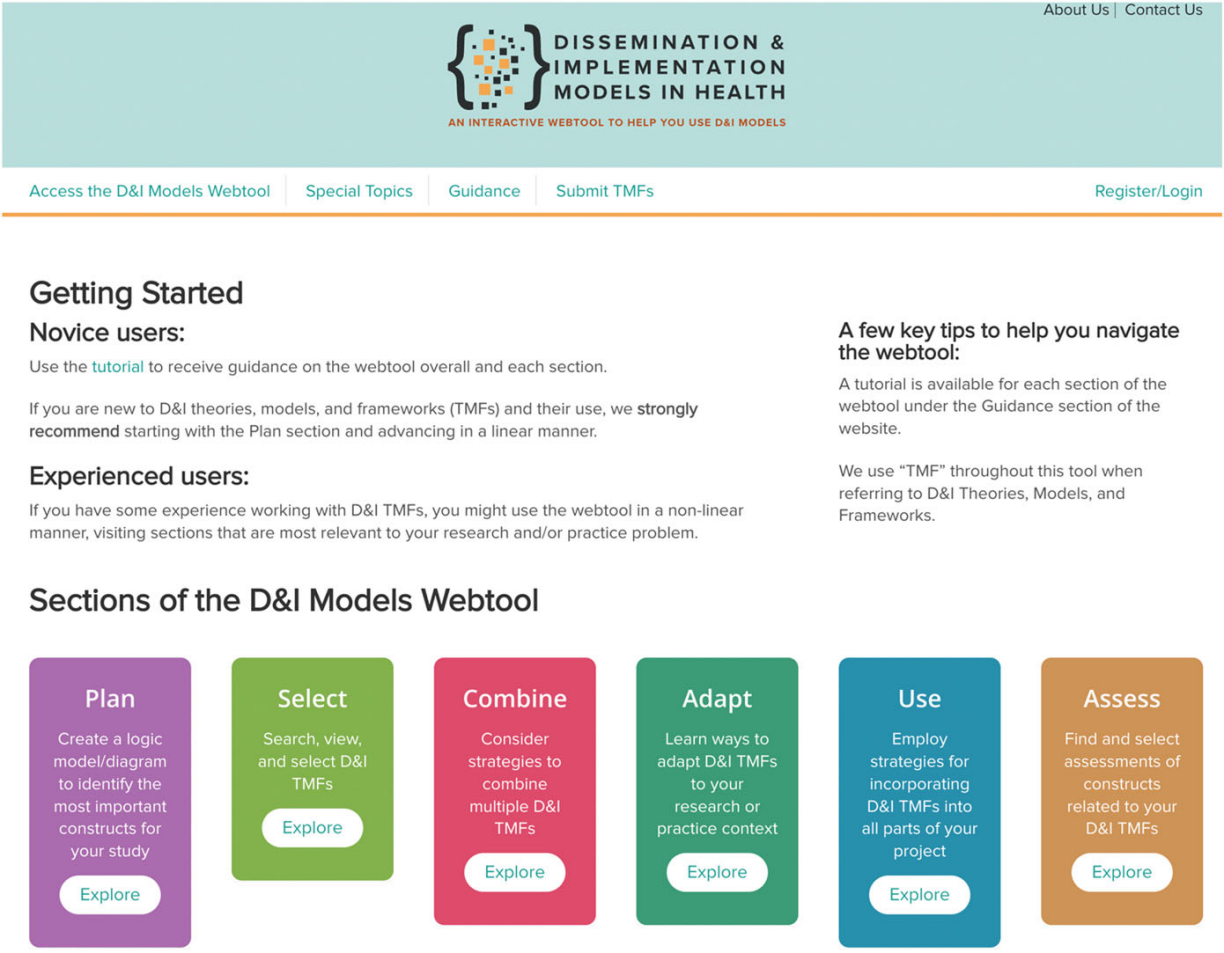
The main sections of the Dissemination and Implementation (D&I) Models in Health webtool. D&I = dissemination and implementation.

The current version of the D&I Models webtool includes a landing homepage, six main sections (i.e., Plan, Select, Combine, Adapt, Use, Assess), the Special Topics section, resource pages (i.e., Tutorial, Glossary, FAQ, Resources) and a link to submit a new D&I TMF. The main sections of the webtool are illustrated in Figure 2 and a brief description of each is provided below.

#### Plan

The Plan section provides an opportunity to create a logic model for one’s D&I project. The logic model is used to identify key constructs for the project, which then aids in model selection in the next section; Select. Constructs are concepts central to a D&I project that will guide the project throughout planning, implementation, and evaluation. Dropdowns reveal a blank logic model worksheet, several completed examples, and videos explaining the planning process. One example illustrates the use of the 5 A’s Cessation Program [13] and it is carried throughout the webtool sections to help users make connections on how the different sections work in tandem.

#### Select

The Select section is designed to help users identify the most appropriate model(s) for their D&I project from among the 110+ D&I TMFs included in the webtool. It contains three subsections that users can navigate flexibly, depending on their level of experience and personal preferences.

The first subsection, Strategies to Select, presents frequently asked questions, such as “Is there one ‘right’ D&I TMF for my project?,” to guide users through key considerations in choosing a model. Concise dropdown responses guide users through the thought process of selecting D&I TMFs before engaging with the following two subsections that list and filter models directly.

**Figure 3.**
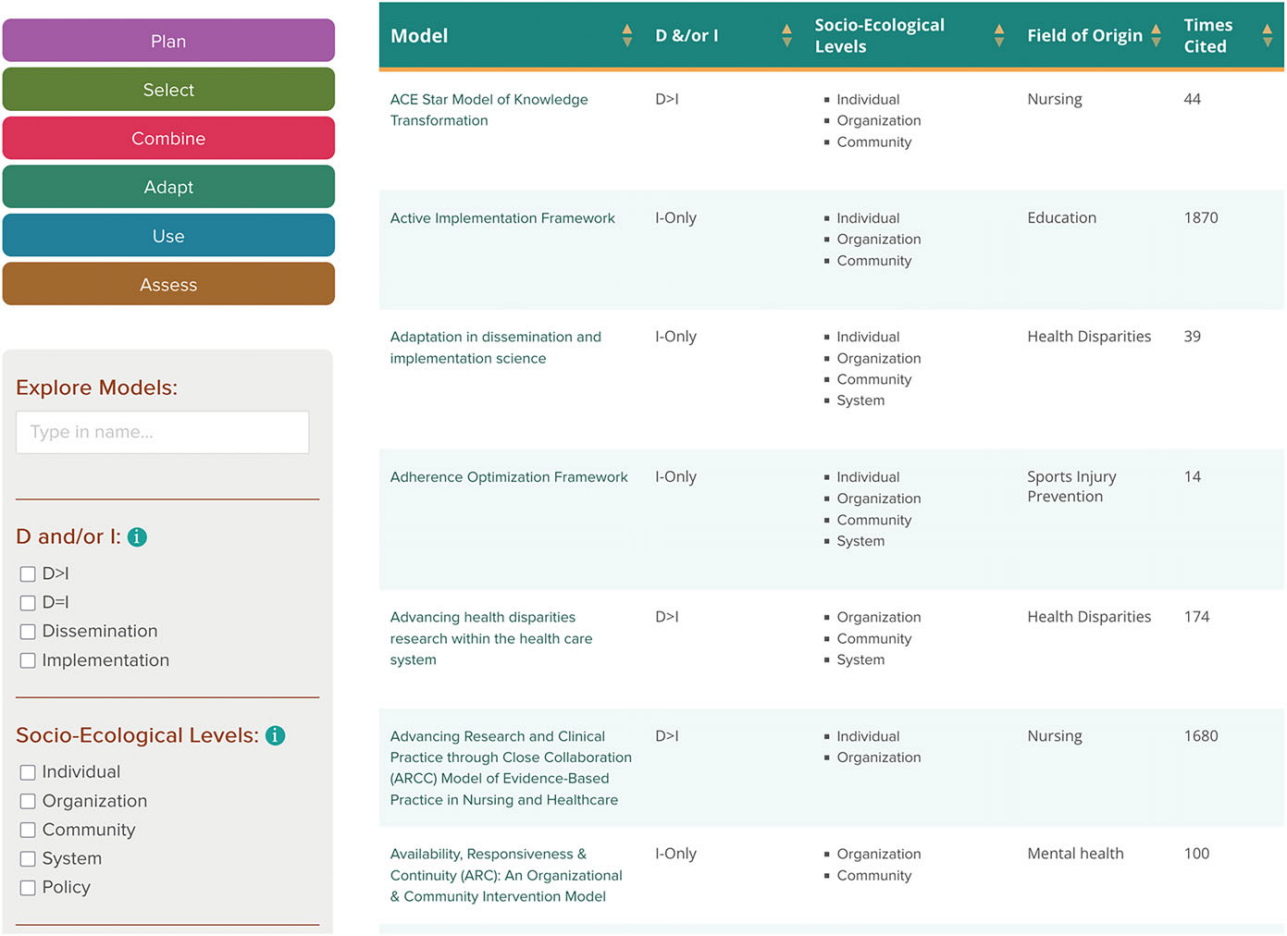
Explore Dissemination and Implementation (D&) Models subsection of the D&I Models in Health webtool. D = Dissemination; I = Implementation.

The Explore D&I TMFs subsection is a core feature of the webtool and serves as the main portal for searching and filtering D&I TMFs (Figure 3). Users can explore D&I TMFs in several ways: by using keywords, checking boxes aligned with specific characteristics (e.g., dissemination vs. implementation focus, socio-ecological level, field of origin), or importing constructs identified during the Planning phase. The search function updates in real time, allowing for rapid filtering and exploration. Search results are returned as a prioritized list based on the number of construct matches, allowing users to assess which models may be the best fit. A summary table highlights the number of construct matches in bold and ranks D&I TMFs accordingly. For convenience, users can export results in CSV or XLSX formats to facilitate further comparison or refinement offline.

Each D&I TMF entry includes a link to a dedicated page providing detailed information such as a D&I TMF description, key constructs, a visual figure (if available), the D&I TMF’s website, original citation(s), published examples of use, and (when available) applications in health equity contexts. Citation counts (updated annually) are included to provide insight into the D&I TMF’s influence in the field. Users can also rate and comment on D&I TMFs through a crowdsourcing mechanism [14,15]. As of June 2^nd^, 2025, there are 117 D&I TMFs linked to 65 constructs and representing 65 fields of origin included in the webtool.

#### Combine

The Combine section provides users with guidance on how to combine multiple D&I TMFs. No one model will likely fit a given D&I project needs perfectly, so users can reference this section to cover all the key constructs and elements within their D&I project thoroughly. This section is organized by common questions related to model combination, such as reasons and first steps on how to combine D&I TMFs. Lastly, we provide citations and links to examples for the successful combination of TMFs. As noted in the Combine section of the webtool, combining D&I TMFs can be advantageous for several reasons. For instance, pairing a determinant framework with an outcome-oriented framework can allow users to leverage the strengths of both (e.g., combining EPIS [16,17] with RE-AIM [18,19] or the Implementation Outcomes Framework [20]. In other cases, researchers may combine frameworks to expand on a specific construct within a broader TMF (e.g., using CFIR [21–23] alongside the Framework for Reporting Adaptations and Modifications-Enhanced [FRAME] [24]) to more fully capture adaptation processes in a multilevel study). Additionally, combining a general D&I TMF – such as PRISM [25,26], EPIS [16,17], or CFIR [21–23] – with a health equity framework [27] can help integrate equity considerations across the implementation process.

#### Adapt

The Adapt section addresses the situation where a D&I TMF does not perfectly fit a D&I project. While the Combine section focuses on using multiple D&I TMFs together to achieve comprehensive coverage, the Adapt section supports users in tailoring the selected D&I TMF(s) to better suit their project needs and context. In this section users can access explanations to questions on when and how to adapt a D&I TMF to fit their project and context better. It also contains a worksheet for users to walk through adaptation considerations in their specific project. An example project worksheet demonstrates model adaptations for the 5 A’s Smoking Cessation Project [13] noted above.

#### Use

The Use section provides guidance on operationalizing the selected D&I TMF(s) throughout a D&I project. Dropdowns provide explanations to commonly asked questions about using D&I TMFs. Our worksheet is specifically designed to help users when writing grant proposals. It walks through how to reference the use of D&I TMFs in each section of a proposal.

#### Assess

The Assess section uses constructs from D&I TMFs and links them to a list of assessment instruments. Each construct is detailed with a link giving a definition, number of D&I TMFs to which it is associated and a list of which D&I TMFs assess the construct.

### New Section On Special Topics

The Special Topics section was created to provide specialized guidance for developing cross-cutting issues on D&I science related to the use of TMFs. The first special topic we created was for health equity due to the large volume of requests our team received to provide guidance on this topic through consultations and the multiple publications encouraging the integration of health equity into implementation science [28–31]. The section includes key definitions for health equity and its relationship with D&I, and D&I TMFs. It also provides a list of health equity dimensions identified in the D&I literature, related definitions, and relevant case examples. Guidance on how to incorporate health equity across and throughout the main sections of the D&I Models webtool (i.e., Plan, Select, Combine, Adapt, Use, and Assess) is also provided. Central to this section are curated case examples using D&I TMFs to address health equity. The initial list of case examples was identified through a scoping and reverse citation review of the literature [32]. A vetted case example template was developed to abstract the most relevant characteristics of D&I TMF use with health equity focus on the selected case studies. The initial set of case examples includes diversity in terms of the D&I TMF, setting, population, health topic, and the health equity dimensions. We anticipate expanding this list with future case examples as new studies are published. A final component of the health equity special topic is a list of annotated references for further reading on the intersection of health equity, D&I, and D&I TMFs.

While conducting this health equity-focused work, we added the construct of “health equity” to the webtool’s *Explore* function detailed above. Each D&I TMF was reviewed and coded, where appropriate, with the new tag, enabling users to find TMFs that purposefully address health equity. Several new TMFs were added to the webtool in this process (e.g., EQ-DI Framework [33], Health Equity Implementation Framework) [27,34].

## Application Guidance and Use Cases for the Webtool

### Guidance

Several pages were created to support navigation of the D&I Models in Health webtool during Phase 1. These include the Tutorial, Glossary, FAQ, and Resources pages. Found in the top navigation bar, the guidance pages remain accessible throughout the user experience for reference and guidance (see Figure 2). Users access the webtool itself through the top navigation or a prominent call to action button, central on the homepage. Further guidance on the webtool landing page provides tips for novice and experienced users, as well as suggestions for a successful user experience.

### Summary of Use Cases

The D&I Models in Health webtool was designed to support both researchers and practitioners with the various activities described below.

*Proposal Writing –* The D&I Models in Health webtool was designed to serve as a resource for those developing a research proposal with substantial D&I science components. Research teams should find the Plan section of the webtool to be an excellent starting point in developing their initial logic model for the proposed research and can also facilitate the refinement of the research questions and identification of key constructs. In doing so, it is recommended that researcher teams include representatives of their key operational, clinical, and/or community partners to create the initial logic model using the worksheet provided. Based on the key constructs identified in the logic model, the Select section can then be used to identify potential D&I TMFs to serve as an overarching D&I TMF for the proposal. After the selection of one or multiple D&I TMFs (further facilitated by the Combine section), D&I TMF(s) can be adapted to the context of the research proposal (see Adapt section) and the Use section can help integrate D&I TMF(s) throughout the proposal. Finally, the Assess section should help to identify assessment approaches and related validated instruments that link to the key constructs of the selected D&I TMFs.

*Consultation –* Consultation is a common way for those new to D&I science to first get exposed to D&I TMFs. The D&I Models in Health webtool can be used before, during, or after consultations to guide the consultee through the Plan and Select sections of the tool and use the results table from the *Explore D&I TMFs* function of the webtool to consider pros and cons of possible D&I TMFs. To make consultations more efficient, we have consultees visit the website prior to a consultation meeting and come with a list of potential D&I TMFs, a logic model, or key constructs that seem most relevant to their project.

*Training and workshops* – Most D&I science trainings emphasize selecting and using D&I TMFs. The D&I Models webtool is well suited to serve as an activity or even a backbone/structure for larger training workshops. The workshop topics can be structured around the main sections of the webtool (i.e., Plan, Select, Combine, Adapt, Use, Assess) and utilize materials and resources (worksheets, examples, references to case examples) provided in each section. For example, our team led a training workshop at the 2019 Annual Conference on the Science of Dissemination and Implementation in Health which followed the structure of the D&I Models webtool at that time and provided real-time linkage to the webtool.

*Courses* – The webtool can be used as part of graduate courses on D&I science (or practice). In our ACCORDS graduate certificate program [32], we have used it as part of several courses and for multiple assignments (https://medschool.cuanschutz.edu/accords/cores-and-programs/dissemination-implementation-core/d-i-certificate-program). It can be an efficient way to have students obtain initial information on a D&I TMF (introductory courses); to develop research logic models (research design and grant writing courses); to identify relevant, validated assessment tools (evaluation courses); and to consider key issues and options in assessing and addressing health equity.

### Usage of the D&I Models in Health Webtool

Since its inception, the D&I Models in Health webtool had a high and increasing number of visits and visitors. Table 1 provides an overview of the visits to the webtool and more detailed summary of use for key sections of the webtool for the last five years (2020–2024).

**Table 1.** 2020–2024 usage statistics – total webtool visits and top 10 most viewed pages on the Dissemination and Implementation (D&I) Models Webtool

Total webtool visit per year	2020	2021	2022	2023	2024
	16,176	23,057	25,871	32,490	37,328
**2024 Statistics by page**					
**Page**	**Pageviews**	**Unique Users**	**Views per User**	**Average Time on Page**	File Download*
Home Landing Page	23,005	15,723	1.46	10s	N/A
Explore D&I TMFs	12,874	5,592	2.30	2m11s	N/A
Select	9,212	4,715	1.95	17s	N/A
Plan	8,551	5,052	1.69	1m10s	5,367
View All D&I TMFs	3,508	336	10.44	4m31s	N/A
Select Strategies	2,492	1,843	1.35	58s	N/A
Health Equity	2,424	1,849	1.31	26s	77
Assess	3,626	1,855	1.95	43s	N/A
Tutorial	2,335	1,664	1.40	1m29s	N/A
Combine	1,873	1,376	1.36	39s	N/A
Adapt	1,754	1,370	1.28	23s	417
Use	1,899	1,411	1.35	27s	513

*Not all pages include downloadable content. D&I = dissemination and implementation. TMF = theories, models, and frameworks.

The most frequently visited pages, aside from the home page, included Explore D&I TMFs (12,874), Select (9,212), and Plan (8,551). Unique users did visit pages multiple times, as indicated in Table 1. When reviewing individual D&I TMF page visits, the D&I TMFs that were visited most frequently included the RE-AIM 1.0 Framework (1,079) [18,19], the Active Implementation Framework (1.061) [35], and the Consolidated Framework for Implementation Research (880) [21 23]. Considering downloaded content, while not every page has available resources to download, the Plan page showed 5,367 downloads, suggesting interest in construct identification and use of a logic model to plan a D&I project as described in the Future Directions here. Several tutorial videos are available on the webtool drawing more than 500 views each in 2024. Average time spent on each page (i.e., time between a person landing on a web page and moving on to another one) varied and longest times were reported for the View all D&I TMFs (4m 31s), Explore D&I TMFs (2m 11s), the Tutorial (1m 29s), and Plan (1m 10s) pages. In terms of geographical distribution of users, in addition to the primarily US-based visits (*n* = 18,835), visitors were also tracked from Canada (*n* = 2,173), multiple European countries (e.g., Netherlands, United Kingdom, and Germany) (*n* = 3,725), Australia (*n* = 1,672), and China (*n* = 1,230).

### Summary and Future Directions

TMFs are core components of D&I research studies, but their selection and proper operationalization can pose challenges for those new to the field. The D&I Models in Health webtool was developed and iteratively improved over the past decade to address this need. It integrates guidance on several key D&I research tasks, such as planning through a logic model, selecting, combining, adapting, and operationalizing D&I TMFs, and linking D&I TMFs with assessment strategies. Through ongoing extensions, additions, and refinements, the D&I Models in Health webtool is evolving into a one-stop-shop for all things related to D&I TMFs. Its extensive and growing use suggests that the webtool is fulfilling this role and continues to be a valued resource in the field.

Additional tools aligning with specific sections of the D&I Models in Health webtool have been developed. We encourage the use of the D&I Models in Health webtool in conjunction with these useful resources to expand specific sections of the webtool. For example, the Implementation Research Logic Model [36] webtool can complement the Plan section, the T-CaST tool [37] which can provide a different classification for comparing D&I TMFs in the Select section, two sets of published worksheets by Moulin and colleagues [38] and more recently by Fontaine and colleagues [39] can further structure the decision process on which models should be selected and steps to operationalize D&I TMFs to complement the Select and Use sections and webpages dedicated to specific D&I TMFs (e.g., Consolidated Framework for Implementation Research (CFIR) [23] and CFIR 2.0 [21,22] which includes the CFIR-ERIC Matching tool, the Exploration, Preparation, Implementation, and Sustainment Model [16,17], or the Reach, Effectiveness, Adoption, Implementation, and Maintenance framework [18,19] and the Practical, Robust Implementation and Sustainability Model [25,26]) to support the operationalization of selected D&I TMFs and expand the Adapt, Use, and Assess sections of the webtool.

The D&I Models webtool was designed to support users with diverse learning needs, levels of experience, and professional backgrounds. To enhance accessibility and usability, we developed tutorial videos, worksheets, and case examples that guide users through selecting and applying D&I theories, models, and frameworks. We also conducted user testing with individuals representing a range of roles and experience levels, and offered live workshops and demonstrations to support learning in interactive settings. These efforts reflect our commitment to making the webtool practical and adaptable for a wide range of learners and will continue as the webtool is refined and expanded.

While the D&I Models in Health webtool has attracted users from a range of geographic areas, the majority of visits have come from English-speaking countries, including the United States and Canada. Expanding its reach and relevance to a more global audience is an important goal for future development. To support broader dissemination and utility, future work will focus on identifying strategic and culturally responsive dissemination approaches that actively engage researchers and practitioners from non-English-speaking countries. Additionally, efforts will be needed to explore how the selection and application of D&I TMFs may differ across diverse cultural contexts, including those rooted in non-Western traditions or in regions outside the Global North, to enhance the accessibility and contextual appropriateness of the webtool.

Our team has several new features planned to be incorporated in the D&I Models in Health webtool in the coming years, depending upon availability of funding. We have prioritized the following expansions:

1) We intend to expand the Special topics with additional key cross-cutting D&I issues. The next three topics we are planning to develop are de-implementation [9], dissemination [40], and HIV/AIDS implementation research.

2) The webtool includes a comment and rate function where users can provide feedback on individual D&I TMFs to support decision-making for other users in selecting appropriate D&I TMFs. This function has been severely underutilized to date. We recently launched a campaign to initiate a crowdsourcing activity to increase ratings and comments for the D&I TMFs.

3) Recently, we started a major redesign and expansion of the Assess section to include direct access and linkage to assessment instruments as a repository along with meta-data variables about each assessment instrument. This work is underway and will be detailed in a separate manuscript upon completion.

## Conclusions

The D&I Models in Health webtool has been widely used, with increasing uptake over time, suggesting it addresses a meaningful need in the field. It offers an improvement over selecting a D&I TMF based solely on prior use, mentor preference, or tradition within a research team.

While the original user interface was functional, it proved suboptimal, particularly for users with less experience, who often struggled with where to begin. In response, we added a Planning section with more concrete guidance, examples, and templates to support navigation and decision-making. Several updates and expansions have followed.

The webtool remains a work in progress and will continue to evolve. Recent upgrades to the platform have made ongoing maintenance more feasible without dedicated developer support, however, larger enhancements require securing additional funding. There is a clear opportunity to formally evaluate the tool’s use under varying levels of facilitation and technical assistance. We welcome user feedback, including suggestions for special topics, new D&I TMF submissions, and comments on applying existing D&I TMFs.
